# Retrospective Impact Evaluation Continuing to Prove Challenging Irrespective of Setting: A Study of Research Impact Enablers and Challenges Cloaked as an Impact Evaluation? Comment on "‘We’re Not Providing the Best Care If We Are Not on the Cutting Edge of Research’: A Research Impact Evaluation at a Regional Australian Hospital and Health Service"

**DOI:** 10.34172/ijhpm.2022.7742

**Published:** 2023-01-24

**Authors:** Shanthi Ramanathan

**Affiliations:** ^1^Hunter Medical Research Institute, Newcastle, NSW, Australia.; ^2^College of Health, Medicine and Wellbeing, University of Newcastle, Newcastle, NSW, Australia.

**Keywords:** Research Impact Evaluation, Australia, Research Investment

## Abstract

The original article provides a detailed and insightful presentation of enablers and detractors for research participation, translation, and impact, at a regional Australian hospital and health service. This information builds on existing knowledge, from the perspective of a non-metropolitan healthcare organisation. It stands to inform all healthcare organisations keen to embed research into their institutions. However, what the article fails to do is present the results of the research impact evaluation in a systematic and useful way for the reader to assess the benefits of research investment by a healthcare organisation including delivery of better quality care and improved patient outcomes. This commentary suggests why such information is critical to justify continued research investment by healthcare organisations and to showcase the potential benefits of the embedded research model. It also discusses the limitations of undertaking impact evaluation retrospectively and suggests that a prospective approach coupled with proper data collection systems and processes upfront could help future reporting of organisational research impact.

 The recent paper by Brown et al^1^ attempted to retrospectively evaluate the impact of research investment from 2008 to 2018 at Townsville Hospital and Health Service (THHS) in Queensland, Australia. A secondary aim was to identify contextual conditions that enable or hinder intended impacts. I commend the authors for their detailed analysis of the contextual factors (such as infrastructure, support, resources, and culture) that enable or hinder both the conduct of research and translation of the findings to achieve intended impacts. Their findings highlight important challenges and enablers for health services operating in rural, regional, and remote settings in Australia (and potentially in other parts of the world) to consider if wanting to champion research as a core function. The study stands to contribute to a growing body of literature on the ongoing challenges of embedding research in healthcare organisations.^2-4^

 However, what the study lacks is a solid contribution to our understanding of the benefits of healthcare organisations investing in research in the first place. Given the current fiscal pressures on the Australian economy on the back of a global pandemic, and the increasing need for institutions and individuals to be accountable for the use of public monies for research, this appears to be the more pressing concern – being able to quantify, qualify, and monetise the returns on all research investment, including investment by healthcare institutions. That is the crux of an institutional research impact evaluation – to evidence and report on the benefits and impacts of the research undertaken by and/or supported by an organisation. It relates directly to Research Question 3 of the study: *What impacts have resulted from the research investment?* Unfortunately, I had to work hard to find any evidence of the impact of THHS’s research investment over the 10-year period. Impact findings were not presented in a manner that gave immediate visibility to the aggregated research impact at the institutional level for THHS. Covering the 10-year period at THHS, only two obvious instances of research outcomes and impacts were found in the article.

 The first was found in Figure 4. This included (*i*) summary statistics for site-specific approvals to conduct the research at various THHS sites and (*ii*) number of publications that were produced from the research. This data was presented to evidence growth in research activity, but neither are accepted indicators or metrics of research impact. Site specific approvals, while important from a research governance and ethics perspective, are a known administrative burden for both researchers and administrators^5,6^ and there is no evidence to suggest they improve the translation of research into policy or practice. Publications are an important output of research and a key translation activity but without any visibility to the usage of these publications evidenced by data such as citation counts, number of reads or downloads, Altmetric scores, or field weighted citation indexes, it is difficult for the reader to evaluate the knowledge impacts (the very lowest order of research impact) from these publications. The paper also points out that the number of professionals undertaking research rose over that 10-year period, therefore an increase in the number of publications would have been expected. So both these statistics, as presented in the article, are measures of research activity and productivity, not impact. More robust indicators such as field weighted citation indexes could have been reported.^7^

 The second place where there was reference to impacts was in Table 2 which presented translational actions within THHS research projects that were reported to deliver clinical, workforce and/or policy impacts. What was immediately highlighted in this table were the translation actions (not really a focus of the paper), rather than the impacts (a key focus of the paper). These translation actions included co-production, choice of research topic/question arising from clinical need, governance infrastructure, multidisciplinary collaboration, communication and policy and professional linkages, as reported by THHS researchers interviewed. Some of these translation actions, for example co-production, have been promoted by funders and interested parties as a means of achieving research impact.^8^ However, the lack of strong evidence of the impact of co-production has led to recommendations for a more cautious approach.^9^ But perhaps what would have improved the reporting was to have highlighted the policy and practice impacts which were “hidden” within this table.

 For example, THHS did not have a policy in skin injuries for neonates. This prompted research on the epidemiology of skin injuries in neonates from mechanical force that, in turn, led to the development of a specific policy and risk assessment tool.^10^ Both these policy and practice impacts were not highlighted in the table. Also, I could not find further data on what these specific policy and practice changes have meant for these neonates, their families, or the health system (eg, better wound management, quicker recovery, earlier discharge)? I would suggest this is a critical component of any research impact evaluation, following the evidence chain and presenting a case study that links the research to delivery of better quality care and improved patient outcomes, a key benefit of embedding research in healthcare. Similarly, what have been the impacts of the Australasian Tele-trial Model for oncology care or the multidisciplinary teleconference approach to prevention and management of limb amputation? These are potentially rich case-studies and there appears to be a missed opportunity to showcase some potential “big wins” for the research investment at THHS using a narrative approach.^11^ A deep dive into these projects would bring to life instances when the embedding of research has led to better care for rural, regional and remote patients in the THHS footprint with potential for translation and scale up to other parts of the country.

 And thirdly, although much less obvious, were a couple of *research capacity building impacts* embedded within the reporting of the qualitative interview data.

 “*TRESA began providing research training in 2016 as a lunch-time series over 12 weeks, or a two-day block mode. This research training… covered aspects such as legislative frameworks, research design and ethics and governance processes. THHS staff attendance at this training increased from 25 in 2016 to 80 in 2018.” *

 In summary, I was not convinced that this paper presented a research impact evaluation using any known methodologies or frameworks of research impact evaluation, of which there are a plethora.^12,13^ Even when using a realist empirical approach which came up with six categories of impact from Phase 1 of the study (ie, research investments; research activity impacts; research capacity impacts; clinical practice and policy impacts; health workforce impacts; and patient and population health impacts) the study failed to present actual impact results within these six categories. For example, although the ability to leverage competitive research funding was listed as an impact in the narrative, no attempt was made to quantify the number of leveraged grants or the value of this leveraged funding.

 This obvious limitation was potentially a result of the retrospective application of impact evaluation to THHS which, amongst other limitations, often suffers from the lack of evidence of impact through common sources like routine data and annual reports. This presents impact evaluators with a dilemma: how to capture and report on the impacts of research when the evidence for the impact pathway is not clear and primary data collection is costly and recall patchy? This limitation has led to the development of frameworks that consider the prospective application of impact to more efficiently and effectively capture impacts of research from the get-go.^14^ While not always practical when having to justify past investment, prospective application of impact evaluation is worth considering for organisations wanting to evidence the impact of their health and medical research on policy, practice, community, society and the economy. It lends itself to setting up cost-effective mechanisms for capturing impact evidence upfront. In this respect, Brown and colleagues are commended for tackling this very issue in the discussion section of their article. They highlight the need for THHS to increase efforts to measure research activity against new metrics in addition to “traditional” research grants and publications metrics. They identified the important data gaps such as:

- The lack of routine, organisational measures of research capacity building despite the well-recognised importance of building research capacity within clinical settings.
- The lack of systematic data collection on clinical practice, policy, workforce and health impacts from research.

They recommend that addressing these gaps in routine data collection needs to be a priority for THHS going forward to enable ongoing assessment of progress towards its research impact goals.

 In closing, perhaps it was the title of the original article that was misleading. The paper was, in my opinion, more about the enablers and barriers to research participation, productivity and impact at a regional Australian hospital and health service, a topic that was well covered. Perhaps a title that reflected this focus would have avoided the confusion.

## Ethical issues

 Not applicable.

## Competing interests

 Author declares that she has no competing interests.

## Author’s contribution

 SR is the single author of the paper.
